# Targeting Stress-Response Pathways and Therapeutic Resistance in Head and Neck Cancer

**DOI:** 10.3389/froh.2021.676643

**Published:** 2021-06-23

**Authors:** Tasia Bos, J. Alex Ratti, Hisashi Harada

**Affiliations:** School of Dentistry, Philips Institute for Oral Health Research, Massey Cancer Center, Virginia Commonwealth University, Richmond, VA, United States

**Keywords:** head and neck squamous cell carcinoma, radiotherapy, resistance, chemotherapy, targeted therapy, oxidative stress

## Abstract

Head and neck cancer is the sixth leading cancer worldwide; head and neck squamous cell carcinoma (HNSCC) accounts for more than 90% of incident cases. In the US, cases of HNSCC associated with human papillomavirus (HPV) have been growing in proportion amongst a younger demographic with superior outcomes to the same treatments, relative to cases associated with tobacco. Yet failures to improve the long-term prognosis of advanced HNSCC over the last three decades persist in part due to intrinsic and acquired mechanisms of resistance. Deregulation of the pathways to respond to stress, such as apoptosis and autophagy, often contributes to drug resistance and tumor progression. Here we review the stress-response pathways in drug response and resistance in HNSCC to explore strategies to overcome these resistance mechanisms. We focus on the mechanisms of resistance to current standard cares, such as chemotherapy (i.e., cisplatin), radiation, and cetuximab. Then, we discuss the strategies to overcome these resistances, including novel combinations and immunotherapy.

## Introduction

### Epidemiology

Head and neck cancer is the sixth most common category of malignancy worldwide, with head and neck squamous cell carcinomas (HNSCCs) accounting for >90% of cases. In much of the world, HNSCC correlates to tobacco usage and is diagnosed in advanced stages. These patients suffer >50% mortality as well as severe disabilities that result from highly toxic multimodality therapy [1–3]. In the developed world, there is rapidly increasing incidence of a human papillomavirus-related (HPV+) subtype of HNSCC, which arises in a younger patient demographic that includes many never-smokers. Worldwide, both HPV(+) and HPV(–) cases typically develop only locoregional disease, with just 7–9% of patients suffering distant metastasis at any point in the disease course. Although they receive the same toxic therapies, the HPV(+) cases have markedly superior survival outcomes [4]; this has created a large population of cured patients with lifelong treatment-related disabilities from a relatively young age and a proportional demand for deintensification strategies.

### Current Standard of Care

Cisplatin remains the favored systemic agent for definitive therapy of HPV(+) and HPV(–) HNSCCs and is combined with external beam radiation during either primary non-surgical therapy or postoperative adjuvant therapy. In both treatment paradigms, cisplatin improves control of locoregional disease but may not prevent the rare instances of distant metastatic relapse, whose incidence was not reduced by the drug in two large phase III adjuvant trials [5, 6]. Cisplatin can cross-link with DNA, most often between guanine-guanine groups, causing DNA damage. This leads to the inhibition of replication and induces the cell death response. Unfortunately, cisplatin has acute dose-limiting toxicities that are potentially lethal and frequently contribute to serious long-term disability [2, 7, 8]. The worst effects of systemic administration include life-threatening bone marrow suppression, irreversible renal injury, and permanent hearing loss. As a result, many patients with poor performance status, reduced renal function, or hearing impairment at baseline suffer worse oncologic outcomes because of contraindication to receiving cisplatin. In addition, cisplatin is well-known to exacerbate the mucositis that is caused by radiation and creates a spectrum of permanent swallowing difficulties, including permanent feeding tube dependence [9, 10]. Thus, there is a major unmet clinical need to avoid the permanently disabling and potentially lethal toxicities of cisplatin for HNSCC patients while maintaining its proven benefit to control locoregional disease. Furthermore, some extent of inherent and acquired resistance to chemotherapy contributes to treatment failure. Therefore, it is necessary to elucidate the mechanisms underlying the development of these resistances.

Radiotherapy (RT) for tumor elimination is based on tumor-targeted ionizing radiation (IR): electromagnetic energy of sufficient magnitude to directly ionize atoms or molecules (e.g., γ-rays) that can induce cell death both directly *via* mitogenic stress and indirectly *via* oxidative stress [11]. Direct ionization of DNA induces double-strand breaks (DSBs) and single-strand breaks (SSBs) that trigger growth arrest and DNA repair. Direct ionization of RNA, lipids and proteins damage cell membranes, cytoskeletal networks and enzyme activity to deregulate cell functions and mitochondrial activity. Direct ionization of water generates reactive oxygen species (ROS)—including superoxide anion, hydrogen peroxide, and hydroxyl radical—that amplifies this damage through electron transfer to DNA, proteins and lipids (i.e., indirect ionization) and dysregulation of endogenous oxidative signaling [12]. Indeed, oxidative stress from RT can produce changes in local tissue electrical parameters (e.g., resistance, impedance module) that are predictive of oral mucositis in HNSCC patients [13]. Nevertheless, after cancer cells are exposed to IR or another source of ROS, numerous cell processes are activated to adapt to the stress and avoid cell death [14].

## Targeting Programmed Cell Death

### TP53

The tumor suppressor TP53 is a transcription factor that regulates the genes to preserve genome integrity in response to diverse endogenous (e.g., DNA damage, ROS, oncogenes) and exogenous stresses (e.g., smoking, cisplatin). At homeostasis, healthy cells maintain a low level of TP53 with continuous production, ubiquitination and degradation; but stresses which damage cell machinery and downregulate the proteasome can suppress the degradation of TP53 and lead to its accumulation. If the degree of stress is relatively low or transient, this accumulation of TP53 activates cell cycle arrest and DNA repair activity for survival. In contrast, relatively severe or sustained stresses lead to unrepairable damage and enhanced accumulation of TP53 that activates its killer functions, including cellular senescence and apoptosis [15, 16].

*TP53* is the most commonly mutated gene (65–85%) in HNSCC, and *TP53* mutations are largely associated with poor survival and resistance to chemotherapy and radiation in HNSCC patients [17, 18]. Thus, the *TP53* status could be a prognostic and predictive biomarker of clinical response in these patients [19]. The exposure to DNA damaging agents (e.g., cisplatin) or radiation induces DNA SSBs or DSBs that activate TP53. In HPV(–) HNSCC cells with loss-of-function mutations in TP53, cell-cycle arrest and apoptosis are mitigated, resulting in cell survival and treatment failure with chemotherapy and radiation [20]. In contrast, gain-of-function (GOF) TP53 (e.g., R248W, R273H, R175H) not only lose the wild-type (WT) TP53 function, but also confer additional oncogenic properties to tumor cells [21–23]. Since most of the GOF TP53 mutations are located at the DNA binding domain, a subset of the target genes of GOF TP53 are different from those of WT TP53. Furthermore, the GOF TP53 protein becomes much more stable than WT TP53. As a result, GOF TP53 leads to resistance to DNA damage-induced cell death *via* downregulation and upregulation of pro-apoptotic and pro-survival genes, respectively. GOF TP53 can also impair recruitment of the Mre11-Rad50-NBS1 (MRN) complex to the site of DNA damage to inactivate ATM and promote proliferation, migration, and invasion, all of which may contribute to resistance [24]. Although HPV(+) oropharyngeal tumors are relatively treatment-sensitive, the mechanism is still obscure [25, 26]. Speculations include an association with TP53 status. The HPV genome contains the oncogenes, E6 and E7, which inactivate TP53 and Rb, respectively. E6 induces TP53 degradation by binding to E6-AP (UBE3A), a ubiquitin-protein ligase, thus, most HPV(+) tumors rarely express TP53 mutations. It has been suggested that E6 levels may be elevated in the early stage of tumorigenesis, so that inactivation of TP53 may be an early transient event [27]. Thus, the remaining WT TP53 may activate the killer functions.

Besides the mutations, the level and function of TP53 is tightly regulated by Mouse Double Minute 2 (MDM2) that is a target gene of TP53 and has an E3 ligase activity. MDM2 is transcriptionally induced by TP53, binds to TP53 and ubiquitinates following the proteasomal degradation. Therefore, TP53 and MDM2 are balanced by a negative feedback mechanism. In HNSCC, overexpression of MDM2 has been reported in ~40% of cases [28]. Mechanistically, it has been demonstrated that nucleotide polymorphisms (e.g., SNP309, rs2279744) contributes to the MDM2 expression, which may determine the sensitivity of chemoradiation [29].

**Figure 1:**
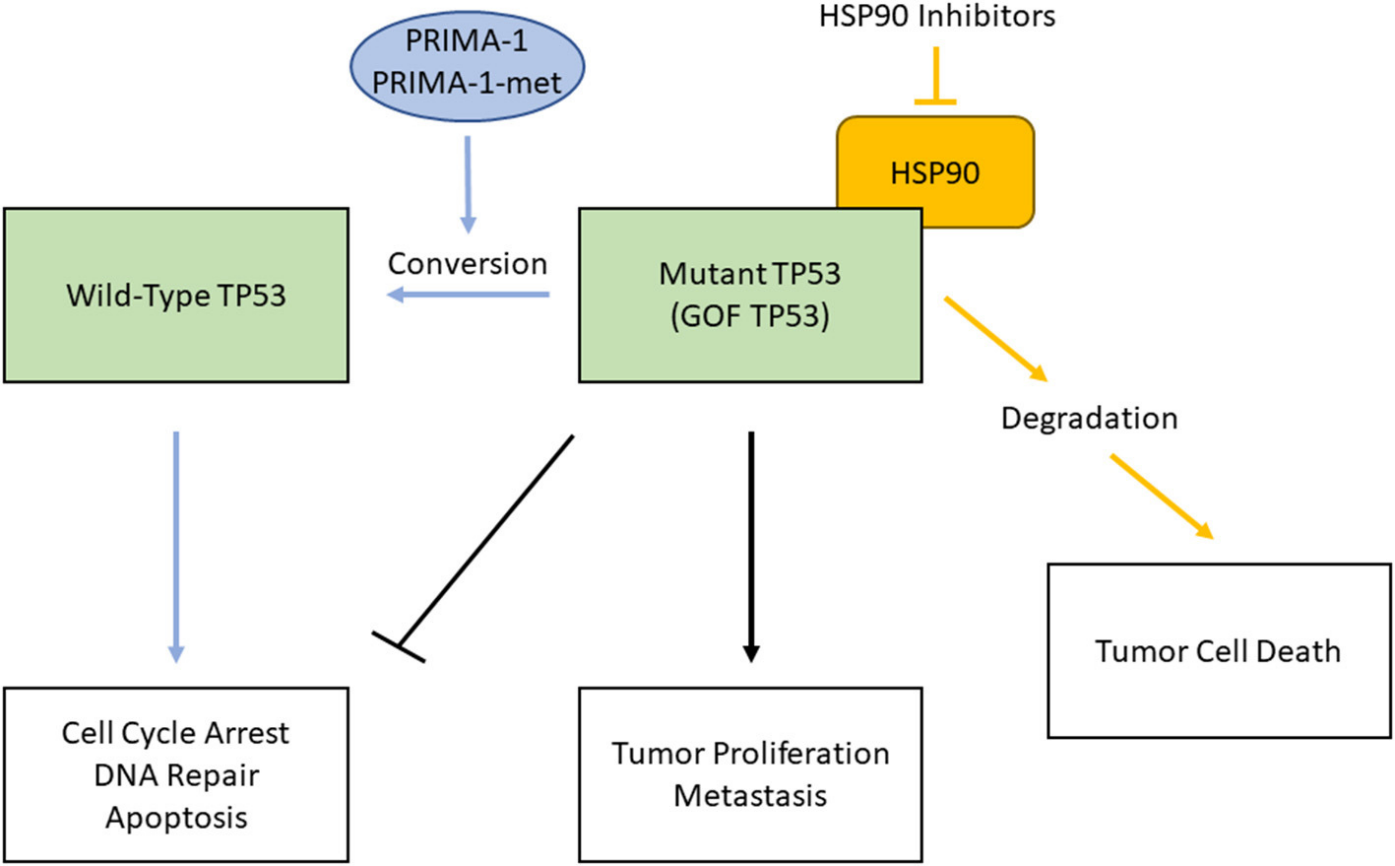
Two strategies to combat therapy-resistance caused by mutant TP53 proteins in HPV(-) HNSCC. Mutant, gain-of-function (GOF) TP53 loses the function of WT TP53 (e.g., cell cycle arrest, DNA repair, apoptosis) and acquires tumor proliferation and metastasis functions (black lines). PRIMA-1 and PRIMA-1-met bind to mutant TP53, interact with the DNA-binding domain, and restore WT TP53 functions (blue lines). Inhibition of HSP90 chaperone machinery destabilizes GOF TP53, which leads to cell death (orange lines).

To combat therapy-resistance conferred by mutant TP53 proteins, there are mainly two strategies to target them either directly or indirectly (Figure 1). WT TP53 is a potent inducer of apoptosis and senescence when expressed in tumor cells, thus reactivation of wild-type function in mutant TP53 is an attractive therapeutic approach to directly target mutant TP53. Since mutant TP53 proteins are generally expressed at high levels, several compounds that can restore WT TP53 function have been developed. PRIMA-1, a mutant TP53-reactivating compound and its derivative, PRIMA-1Met (APR-246), specifically bind to mutant TP53 proteins and interact with the DNA-binding domain, thereby promoting proper folding of mutant proteins and restoration of some WT TP53 functions [30, 31]. While APR-246 is being investigated in the Phase III clinical trial with TP53 mutant myelodysplastic syndromes (NCT03745716), one proposed mechanism of action is that both compounds are converted to methylene quinuclidinone (MQ) to covalently bind and modify thiols in the TP53 core domain, thus causes TP53 to restore its function to induce tumor cell death [32].

Since GOF TP53 is often highly stabilized by the chaperone machinery, targeting this machinery is another strategy that induce mutant TP53 inactivation through protein degradation. For example, a major determinant of mutant p53 stabilization is mediated by HSP90 machinery. Inhibition of HSP90 alone or in combination with its regulator, HDAC6, a cytosolic non-histone histone deacetylase (HDAC), has marked anti-tumoral effects *in vivo* [33–35]. Inhibition of HDAC6 leads to inactivation of HSP90 and allows for reactivation of ubiquitin ligases MDM2 and CHIP E3 to mediate mutant TP53 degradation, but not degradation of WT TP53 [33]. These anticancer effects are concomitant with mutant TP53 degradation followed by cancer cell death, indicating tumor addiction to highly stable mutant TP53.

### The BCL-2 Family

The BCL-2 family primarily regulates mitochondrial-dependent apoptosis induced by a variety of external stimuli including chemotherapy and radiation. The BCL-2 family is subdivided into three groups based on the structure and function; [1] multi-domain pro-apoptotic (e.g., BAX, BAK), [2] pro-survival (e.g., BCL-2, BCL-X_L_, MCL-1), [3] BH3-only pro-apoptotic (e.g., NOXA, BIM, BAD, BID). When BH3-only proteins are activated by a variety of external stimuli, BAX and/or BAK is subsequently conformationally changed and oligomerized at the mitochondria, resulting in cytochrome c release to the cytosol, while the pro-survival proteins prevent the activation of BH3-only proteins and/or BAX/BAK [36–38]. The balance between pro- and anti-apoptotic proteins governs the cells either to survival or death. Tumor cells often overexpress anti-apoptotic proteins, such as BCL-2, BCL-X_L_ and MCL-1, which results in the intrinsic as well as the acquired resistance to chemotherapy and radiotherapy. BCL-X_L_ and MCL-1 are overexpressed in a majority of primary HNSCC specimens, whereas overexpression of BCL-2 is observed somewhat less frequently [39]. Upregulation of BCL-2 and BCL-X_L_ is correlated with chemotherapy-resistance in HNSCC cells, and downregulation of these proteins by antisense or siRNA can sensitize to chemotherapy in HNSCC cells. It has been reported that BCL-X_L_ is involved in the resistance of oropharyngeal cancer to ionizing radiation, and radioresistant laryngeal cancer is associated with BCL-2 expression [40]. Another report suggests that MCL-1 expression (but not BCL-2 or BCL-X_L_) is upregulated in both chemo-resistant HNSCC lines and chemo-resistant tumors compared with chemo-sensitive counterparts [41]. These data suggest that these anti-apoptotic proteins confer chemotherapy and radiotherapy resistance.

**Figure 2:**
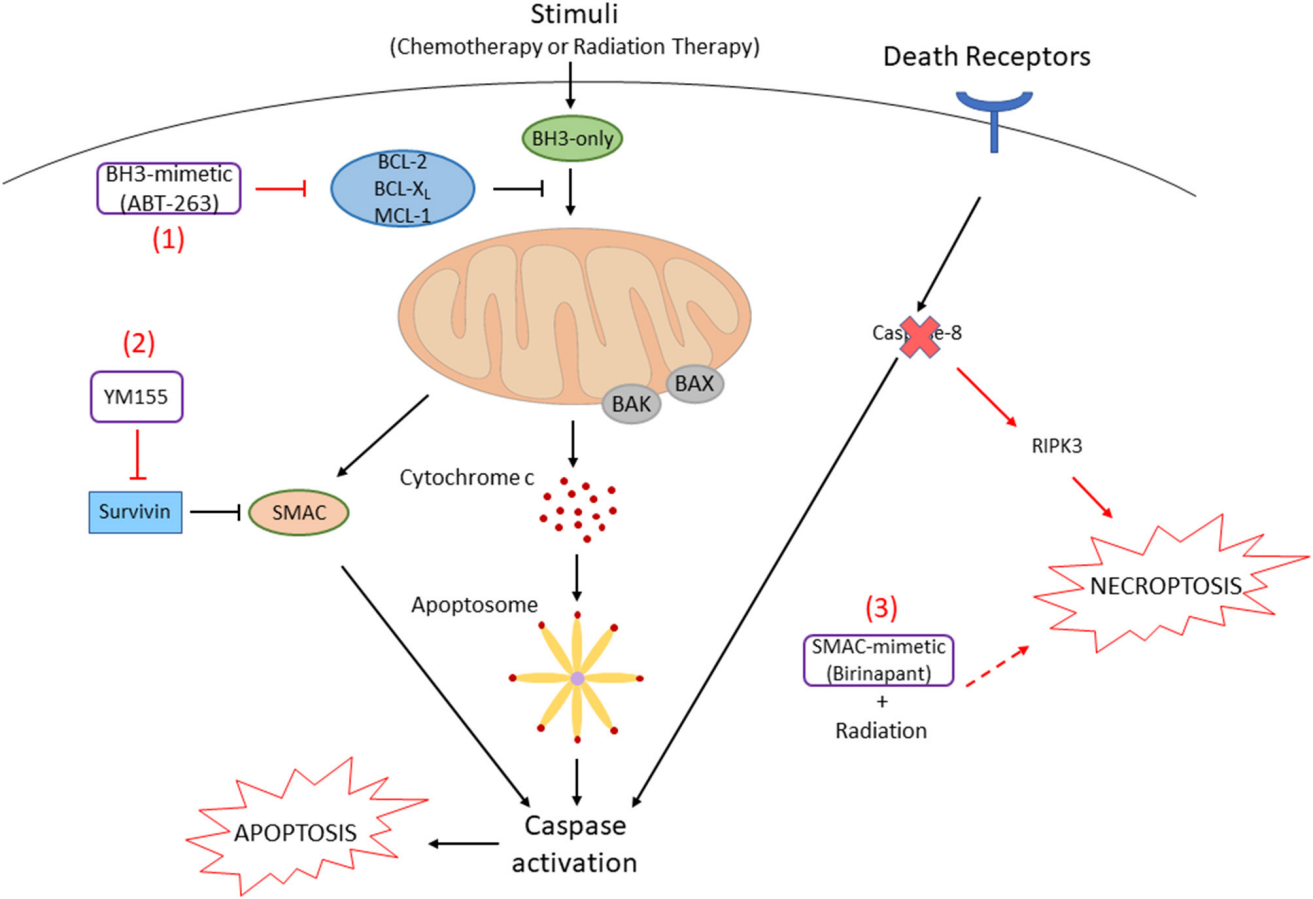
Small-molecule inhibitors promote cell death in HNSCC cells. (1) In HNSCC cells, anti-apoptotic BCL-2 family proteins are often overexpressed. The BH3-mimetic compound, ABT-263, inhibits the function of these proteins by binding to their hydrophobic pockets. Pro-apoptotic BCL-2 family proteins (BAX, BAK) release to initiate the release of cytochrome c, following downstream caspase cascades that result in apoptosis. (2) Survivin, an Inhibitor of Apoptosis Protein (IAP), inhibits Smac that is released from the mitochondria followed by cell death stimuli. Survivin is also often overexpressed in HNSCC, which can be inhibited by the survivin inhibitor, YM155. (3) Caspase-8, which is activated by extrinsic death receptors (e.g., FAS, TNFR) to induce apoptosis, is mutated and inactivated in ~10% of HNSCC. When caspase-8 is inactivated, RIPK3-mediated necroptosis can be promoted. Necroptosis by radiation is enhanced when combined with a SMAC mimetic, birinapant.

In order to inhibit the function of anti-apoptotic BCL-2 family members to overcome resistance, a number of small molecules have been developed. These compounds, so called BH3-mimetics, bind to the hydrophobic pockets of anti-apoptotic BCL-2 family proteins, which results in the release of pro-apoptotic BCL-2 family proteins to activate downstream caspase cascades [42, 43] (Figure 2). ABT-737 is a prototype of this class of compound that binds to BCL-X_L_ and BCL-2, resulting in the release of pro-apoptotic BH3-only proteins and BAX/BAK. This compound can strongly synergize with the chemotherapy drugs or radiation to promote apoptosis of HNSCC cells [44, 45]. More recent reports have shown that inhibition of BCL-X_L_ by an orally available ABT-737-derivative, ABT-263 (navitoclax), and MCL-1 by A-1210477 enhances apoptosis in HNSCC cells [46]. We have recently demonstrated that simultaneous inhibition of all anti-apoptotic BCL-2 family proteins by ectopic expression of NOXA, a BH3-only protein inhibiting MCL-1, and ABT-263 enhances apoptosis regardless of the HPV or p53 statuses [47]. Furthermore, an inducer of endoplasmic reticulum stress, fenretinide, increases the NOXA expression, and a combination of fenretinide and ABT-263 strongly enhances apoptosis in HNSCC cell lines. Future clinical trials using the above drugs in combination are awaiting to judge efficacy and non-specific toxicities.

### Survivin

Survivin was originally identified as an Inhibitor of Apoptosis Protein (IAP) family member that acts as a cell death suppressor [48]. Subsequently, survivin has been recognized as a mediator between the cell cycle and apoptosis [49]. Survivin can block apoptosis and promote cell proliferation and survival by the interaction with the proteins that regulate intrinsic and extrinsic apoptotic pathways. Cytosolic survivin prevents caspase-9 activation by binding to Smac (second mitochondria-derived activator of caspase) and prevents subsequent caspase-3 activation [50]. Survivin also inhibits caspase-independent cell death by interacting with the apoptosis-inducing factor (AIF) [51]. In addition, survivin contributes to the cellular stress response by interfering with autophagy [52]. For example, Beclin-1 is able to bind to survivin, resulting in the initiation of autophagy. In addition, survivin interacts with LC3 (microtubule-associated protein 1 light chain 3) and interferes with the formation of autophagosome by preventing the conversion from LC3-I to LC3-II [53].

Survivin is often overexpressed in about 80% of HNSCCs and 50% of premalignant lesions, suggesting that survivin may be involved in the early stages of tumorigenesis [54, 55]. Survivin is proposed as an ideal biomarker for HNSCC due to its expression in selective tumors and near absence in normal tissues [56]. Interestingly, the localization of survivin in cytosol is associated with poor overall survival and disease outcome [57, 58]. Furthermore, it has been demonstrated that cytosolic survivin confers resistance to chemotherapy- and radiation-induced apoptosis in HNSCC cell lines [59, 60].

A selective small-molecule inhibitor for survivin, YM155, has been developed, and it can reverse cisplatin-resistance by decreasing the levels of cytoplasmic survivin [61]. YM155 can induce apoptosis through mitochondrial- and death receptor-dependent pathways (Figure 2). YM155 can also induce autophagic cell death in HNSCC cells by inhibiting the pro-survival AKT/mTOR pathway and inducing Beclin-1 expression [62]. YM155 is able to promote autophagic cell death in breast cancer cells by increasing the conversion from LC3-I to LC3-II [63]. Phase I/II clinical trials have investigated the effect of YM155 in patients with advanced solid tumors including HNSCC [64–66]. The trials revealed that YM155 as monotherapy is safe with slight side effects, but positive effects are not observed in patients. It may still have a clinical benefit in combination with a less toxic dose of chemotherapy, radiation, and other targeting drugs.

### Caspase-8

Caspase-8 (CASP8) is an initiator of the extrinsic apoptosis pathway and inhibits necroptosis, indicating a molecular switch for these cell death pathways [67]. In HPV (-) HNSCC, caspase-8 is one of the most frequently mutated genes, with approximately 10% of cases [68]. The distribution of *CASP8* mutations in patient tumors and cell lines suggests that the function of mutants is inactivated. The loss of caspase-8 function is known to shift signaling by death receptors from apoptosis signaling to necroptosis signaling. SMAC mimetics are small-molecule inhibitors that generally promote caspase activation and apoptosis through neutralization of IAPs [69]. Several preclinical studies have demonstrated that SMAC mimetics enhance radiosensitivity in HNSCC xenograft models. It has been recently demonstrated that inhibition of CASP8 function enhances necroptosis by radiation when combined with a SMAC mimetic, birinapant *in vitro* and *in vivo* (Figure 2) [70]. Furthermore, the level of RIPK3 expression determines necroptosis sensitivity in HNSCC. Thus, the status of CASP8 and RIPK3 would be biomarkers to justify the necroptosis pathway as a therapeutic target in HNSCC patients.

## Targeting Cell Machinery that Self-Heals Stress: DNA Repair and Autophagy

### DNA Repair

Several forms of DNA damage have been shown to activate TP53, including those generated by chemotherapy and radiation. Since chemotherapy- and radiation-induced DSBs are primarily repaired *via* non-homologous end joining (NHEJ), targeting NHEJ has the potential to sensitize tumor cells to chemotherapy and radiation [71, 72]. NHEJ repair consists of termini recognition, bridging, processing, and ligation of DNA. For example, Ku80 expression is associated with locoregional failure and patient death post radiotherapy in HNSCC [73]. It has been shown that depletion of Ku70 or Ku80 sensitizes pancreatic cancer cells to radiation [74]. Although there are currently no Ku inhibitors, inhibition of Ku proteins with concurrent radiation offers an attractive treatment option, and Ku could serve as a DNA repair-related biomarker of radioresistance in HNSCCs [73]. DNA-dependent protein kinase (DNA-PK) is responsible for phosphorylation of key proteins required for the NHEJ pathway, and when it is inhibited, repair is compromised. Thus, several DNA-PK inhibitors have been developed [75, 76]. In addition, compounds for targeting DNA end-processing have been made to disrupt NHEJ repair following radiation-induced DSBs. Inhibitors of DNA ligase IV have been developed to inhibit the ligation step of NHEJ repair [77, 78].

In response to DNA damage, TP53 activation leads to cell cycle arrest and initiation of DNA repair. Thus, inhibitors of the DNA damage response (DDR) and cell cycle progression have potential to sensitize to chemotherapy and radiation in HNSCC cells with loss of function TP53. This therapeutic vulnerability has been exploited by targeting the cell cycle using small-molecule inhibitors for ATM, ATR, the checkpoint kinase-1/2 (CHK1/2), and the WEE1 kinase [79–83]. The ATR-ATM-CHK1-WEE1 signaling pathway is crucial for the surveillance mechanism of the replication phase and is activated at low thresholds even during the unperturbed S-phase [84–86]. Therefore, cells with replication stress can undergo senescence or apoptosis through inhibition of this pathway. Several inhibitors of this pathway in combination with chemotherapy or radiation have been going into clinical trials with a variety of solid tumors including HNSCC [18].

### Autophagy

Autophagy is a cellular recycling and quality control mechanism required for eliminating unnecessary or non-functional cellular organelles and proteins in living cells. Although the basal level of autophagy is thought to contribute to cell survival in cancer cells, prolonged activation of autophagy can cause cell death by self-degradation of cellular components [87]. Thus, autophagy is considered as a double-edged sword in tumorigenesis and resistance to treatments. In radioresistant cells, anti-apoptotic BCL-2 protein is often overexpressed, leading to inhibition of apoptosis [88]. Since Beclin-1 has a BH3-domain and is capable of binding to BCL-2, Beclin-1-dependent cytotoxic autophagy can be inhibited, which may maintain a basal level of autophagy for cell survival, rather than cell death [89]. Moreover, suppression of autophagic cell death, but not apoptosis, might be a reason behind radio-resistance, and thus, prolonged enhancement of autophagy may significantly sensitize to radiotherapy in such tumor cells [90].

On the contrary, cell survival activity of autophagy results in chemoresistance to DNA damaging agents such as cisplatin in several cancers [91, 92]. Moreover, it has been shown that hypoxia suppresses the cytotoxic autophagy activation toward a pro-survival mechanism by inhibiting autophagy-mediated cell death, as observed in HNSCC cells treated with etoposide [87]. In such cases, inhibition of autophagy either by using pharmacological inhibitors or by siRNA-mediated inhibition has shown favorable treatment outcome.

Cancer-associated fibroblasts (CAFs) play important roles in tumorigenesis of tongue squamous cell carcinoma (TSCC). It has been demonstrated that CAFs confer cisplatin resistance of TSCC cells through autophagy activation [93]. Inhibition of autophagy by chloroquine or Beclin-1 siRNA in TSCC cells can increase cisplatin-induced apoptosis and inhibit viability of TSCC cells co-cultured with CAFs, suggesting that autophagy inhibition could be a strategy to overcome chemoresistance of TSCC. In contrast, New et al. [94] demonstrated that CAF-facilitated HNSCC progression can be reduced after blocking CAF autophagy. The levels of secreted IL-6, IL-8, and other cytokines in cell growth-conditioned media were modulated by blockade of CAF autophagy with chloroquine or Beclin-1 siRNA. When HNSCC cells are cocultured with normal fibroblasts, autophagy is upregulated through IL-6, IL-8, and bFGF (basic fibroblast growth factor). In a mouse xenograft model of HNSCC, the inhibition of autophagy with a Vps34 inhibitor, SAR405, enhanced the antitumor efficacy of cisplatin. These results strongly suggest that inhibition of CAF autophagy could overcome cisplatin-resistance of HNSCC.

## Targeting Cell Transformation that Escapes Stress: Cancer Stem Cells and Epithelial-Mesenchymal Transition

Ample evidence demonstrates that a small fraction of cells serve as CSCs or cancer initiating cells that are critical for tumor initiation and growth and might be associated with metastasis and tumor recurrence [95, 96]. It has been hypothesized that CSCs are responsible for tumor recurrence or resistance after chemotherapy. Epithelial to mesenchymal transition (EMT) is a process that allows epithelial cells to acquire mesenchymal properties to become migratory and invasive. This is linked to aggressive disease progression in multiple cancers and allows tumor cells to escape apoptosis. The role of EMT in HNSCC has largely not been elucidated, but several studies suggests EMT as a prognostic marker [97]. In HNSCC, TWIST1, a basic helix-loop-helix transcription factor, and BMI1, a polycomb-group protein which regulates gene transcription, act cooperatively to induce EMT and stemness, thereby indicating a role for BMI1 in HNSCC metastasis. TWIST1 has been implicated in resistance to both cisplatin and cetuximab [98]. BMI1+ CSCs drive invasive growth and cervical lymph node metastasis in squamous cell carcinoma. BMI1+ CSCs have increased AP-1 activity and are cisplatin-resistant, and combination therapy that targets BMI1+ CSCs and the tumor bulk yields better outcomes and effectively prevents metastasis [99]. Because high fatality rates in HNSCC are mainly caused by metastases, the use of therapeutics to target EMT has potential to provide better prognosis. A study explored CSC-3436, a flavonoid derivative, to inhibit TWIST-induced EMT, metastases, and tumor-initiated ability through the TWIST/BMI1-Akt/β-catenin pathway [100]. HNSCC patients without TWIST1 or BMI1 expression have a better prognosis compared to patients that express both proteins. Therefore, it is warranted to develop a drug that can target TWIST1 and BMI1 for HNSCC treatment.

## Targeting Immunomodulation that Clears Damaged Cells and Adapts to Chronic Stress

Chemotherapy and radiation augment antigen-specific antitumor immune responses. There are various mechanisms involved in this process including; [1] activation and proliferation of tumor-infiltrating lymphocytes, [2] altering chemokines that preferentially recruit cytotoxic T lymphocytes and lead to the upregulation of major histocompatibility complex (MHC) class I expression, [3] release of tumor neoantigens through inflammatory cell death, [4] activation and migration of dendritic cells. Of note, an abscopal effect with higher doses of irradiation in one area results in tumor regression outside the field of radiation [101, 102]. Therefore, numerous clinical trials are ongoing in chemotherapy or radiation in combination with immunotherapy for HNSCC.

Radiation can alter the expression of cell adhesion molecules and increase the density and infiltration of tumor-infiltrating lymphocytes (TILs) involved in tumor cell lysis. For instance, the expression of cell adhesion molecules (e.g., ICAM-1) on the cell surface of endothelium are enhanced by radiation [103]. TILs are emerging as biomarkers in HNSCC as certain TILs such as CD3, CD4, and CD8 T cells are associated with improved prognosis and therapy response. A study observed that HNSCC patients with high expression of CD3 and CD8 T cells in their tumors benefited from longer overall survival, distant-metastasis-free survival, and progression-free survival compared to the patients with low expression of these TILs [104]. Another study reported that HNSCC patients with CD4 and CD8 T cells were associated with improved overall survival [105]. It is also important to note that patients with HPV(+) HNSCC are reported to have higher levels of TILs thus are characterized with longer survival.

Radiation can induce immunogenic cell death (ICD) through damage-associated molecular patterns (DAMPs) in cancer cells. For example, radiation-induced high mobility group protein B1 (HMGB1), a ligand for TLR4, activates the innate immune response and changes the cytokine profile toward an immune stimulatory phenotype [106]. Radiation can also activate antigen-specific anti-tumor immune responses through the upregulation of MHC class I expression [107]. Chemotherapy can increase antigen presentation, leading to increased T cell activation [108, 109]. Furthermore, chemotherapy increases the cytotoxic effects of CTLs and induce ICD [110, 111]. It has also been demonstrated that cisplatin, taxanes, and 5-fluorouracil (5-FU), which are frequently used for HNSCC treatment, decrease myeloid derived suppressor cells (MDSCs) in animal models, which can ultimately enhance anti-tumor immunity [112–114]. Moreover, platinum compounds can increase T-cell activation by dendritic cells (DC) through downregulation by the STAT6 pathway [115].

Based on the diverse immunomodulatory effects of chemotherapy and radiation, the combination of chemo/radiotherapy and immunotherapy is under intensive investigation [102]. Programmed death-1 (PD-1) and its ligand, PD-L1, are immune checkpoints that are exploited in HNSCC to for tumor immune invasion. The anti-PD-1 antibodies, pembrolizumab and nivolumab, were FDA-approved for HNSCC in 2016. These drugs are designed to enhance antitumor immune activity by blocking the PD-1/PD-L1 pathway. Currently, numerous clinical trials are testing the potential synergistic combination of anti-PD-1/PD-L1 with radiotherapy and platinum or cetuximab, or anti-PD-1/PD-L1 used in a neoadjuvant setting [116–118].

HNSCC can develop as a response to chronic inflammation. Inflammatory chemokines, cytokines, and growth factors can contribute tumor proliferation. Chronic inflammation can induce MDSCs, regulatory T cells (Tregs), and tumor-associated macrophages (TAMs), which can impair T cell response, resulting in tumorigenesis while chronic inflammation can suppress T cells and NK cells. The inflammasome, NLRP3 (nod-like receptor protein 3), can initiate inflammatory cell death and promotes tumorigenesis in HNSCC. Studies have demonstrated that NLRP3 is overexpressed in human tissues and that NLRP3 initiates the release of inflammatory cytokines such as IL-1β and IL-18, which are also associated with HNSCC tumorigenesis. When NLRP3 was inhibited in mice, MDSCs, Tregs, and TAMs were decreased, and 5-FU-induced apoptosis was enhanced [119, 120]. Thus, NLRP3 would be a potential target to overcome chemo-resistance.

Dendritic cells (DCs) are the most potent antigen presenting cells and are essential to initiate a primary immune response and migrate to lymphoid organs to present processed antigens to lymphocytes and stimulate immune response. DCs are known to infiltrate solid tumor tissues including HNSCC. EGFR-targeted cetuximab or nimotuzumab antibody dependent cellular cytotoxicity (ADCC) and activated NK cells were both shown to induce natural killer (NK)-DC crosstalk, which resulted in DC maturation and EGFR-specific CD8(+) T-cell priming and enhanced anti-tumor effects [121, 122]. Cetuximab-activated NK cells were also shown to secrete the cytokines IFN-γ, MCP-1, MIP-1β, which inhibited tumor cell proliferation, enhanced antigen presentation, and chemokines that aid in the chemotaxis of T cells. Therefore, a potential therapeutic strategy involves combining cetuximab with an immune cell targeting mAb. CD137, a member of the tumor necrosis factor receptor family, is activated in CD8(+) T cells, NK cells, and DCs. Urelumab, CD137-specific human IgG4 mAb, enhances the efficacy of cetuximab in head and neck cancer patients by enhancing NK-DC crosstalk and inducing DC maturation [123, 124].

## Targeting the Oncogenic Pathways that Drive Constitutive Stress for Survival

### Epidermal Growth Factor Receptor

**Figure 3:**
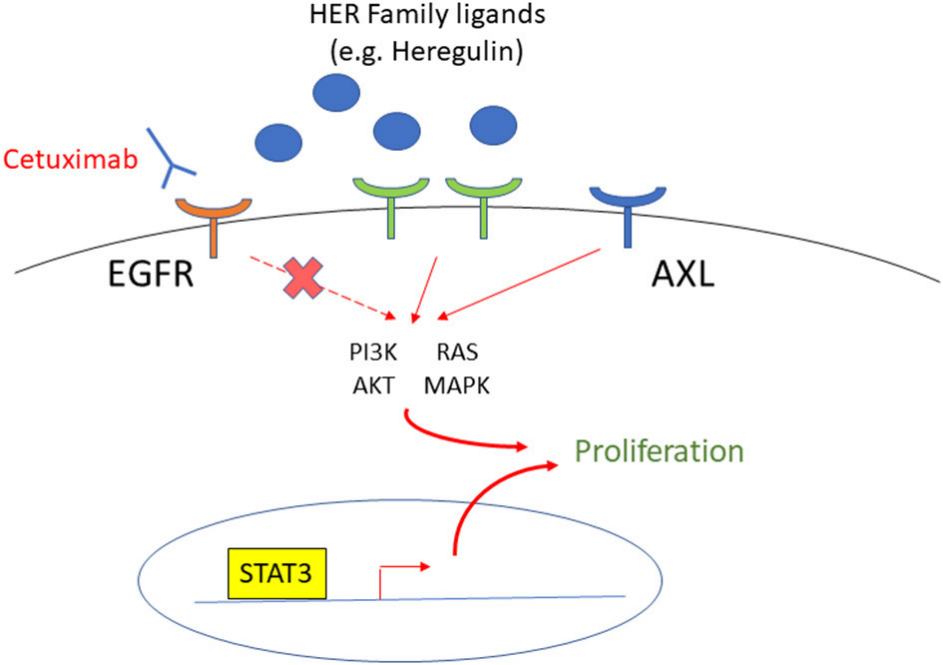
The targets to overcome cetuximab resistance in HNSCC patients. EGFR is overexpressed in most HNSCC, and cetuximab is an FDA approved monoclonal antibody to target EGFR. The HER Family, AXL, and STAT3 are upregulated in cetuximab-resistant HNSCC, and therefore, are targets to overcome cetuximab resistance.

Overexpression of epidermal growth factor receptor (EGFR) is commonly associated with tumorigenesis and treatment resistance in HNSCC. EGFR is overexpressed in 90% of HNSCCs and as a consequence, two primary downstream pathways, PI3K/Akt/mTOR and RAS/RAF/MEK/ERK, are activated and contribute to cell proliferation and survival [125]. Cetuximab, a monoclonal antibody targeting EGFR, is one of the few drugs approved by FDA for use in HNSCC, but its response rate is only 13% when used as monotherapy [126] (Figure 3). It is now well-acknowledged that EGFR amplification or overexpression cannot be used for the prediction of cetuximab response in HNSCC.

A recent proteogenomic study of 108 HPV(–) HNSCC patient tumors suggests that the EGFR ligands, instead of the receptor itself, are the rate-limiting factors for EGFR pathway activity [127]. For example, heregulin, a ligand of the epidermal growth factor receptor 3 (HER3) of the same family as EGFR, is upregulated in HNSCC cells with acquired cetuximab resistance and confers survival through the AKT signaling. However, cell growth is decreased by Pan-HER inhibition by the small molecule tyrosine kinase inhibitor (TKI), afatinib, which inhibits EGFR, HER2, HER4, and signal transduction of HER3 [128], suggesting that targeting the HER family effectively overcomes resistance to cetuximab. In addition, the receptor tyrosine kinase AXL has been implicated in cancer cell resistance to anti-EGFR TKI [129]. Since AXL is upregulated in both acquired and intrinsically cetuximab-resistant HNSCC cells, this is one of the targets that show the greatest promise for overcoming resistance to cetuximab. Another target of interest is signal transducer and activator of transcription 3 (STAT3). STAT3 has been shown to be upregulated and more active in cetuximab-resistant HNSCC cell lines, and its inhibition decreased cell growth in cell lines resistant to anti-EGFR therapy [130, 131].

### The Rb Pathway

Cyclin-dependent kinases (CDKs) are critical regulators of cell cycle progression. The key regulatory pathway of the G1-S transition is cyclin D–CDK4/6–INK4–Rb. When activated by mitogenic signals, both CDK4 and CDK6 kinases can associate with three cyclin Ds (D1, D2 and D3). These active complexes of cyclin D–CDK4/6 phosphorylate and inactivate the retinoblastoma protein (Rb), which promotes the dissociation of the Rb–E2F complex repressive in terms of E2F-dependent transcription. The released E2F then activates the genes necessary for entry into the S phase and DNA replication. The CDK4/6 activity is negatively regulated by p16 INK4A. INK4A inhibits the cyclin D–CDK4/6 activity by directly binding to the CDKs. While loss of Rb is responsible for the G1–S transition in certain types of cancer, the vast majority of cancers have wild-type Rb. These Rb-positive cancers may develop by overexpression or activation of cyclin D–CDK4/6, by loss of negative regulators of cyclin D-CDK4/6 (e.g., INK4A), or by oncogenic signaling pathways that activate cyclin D–CDK4/6 for cell proliferation [132–134].

**Figure 4:**
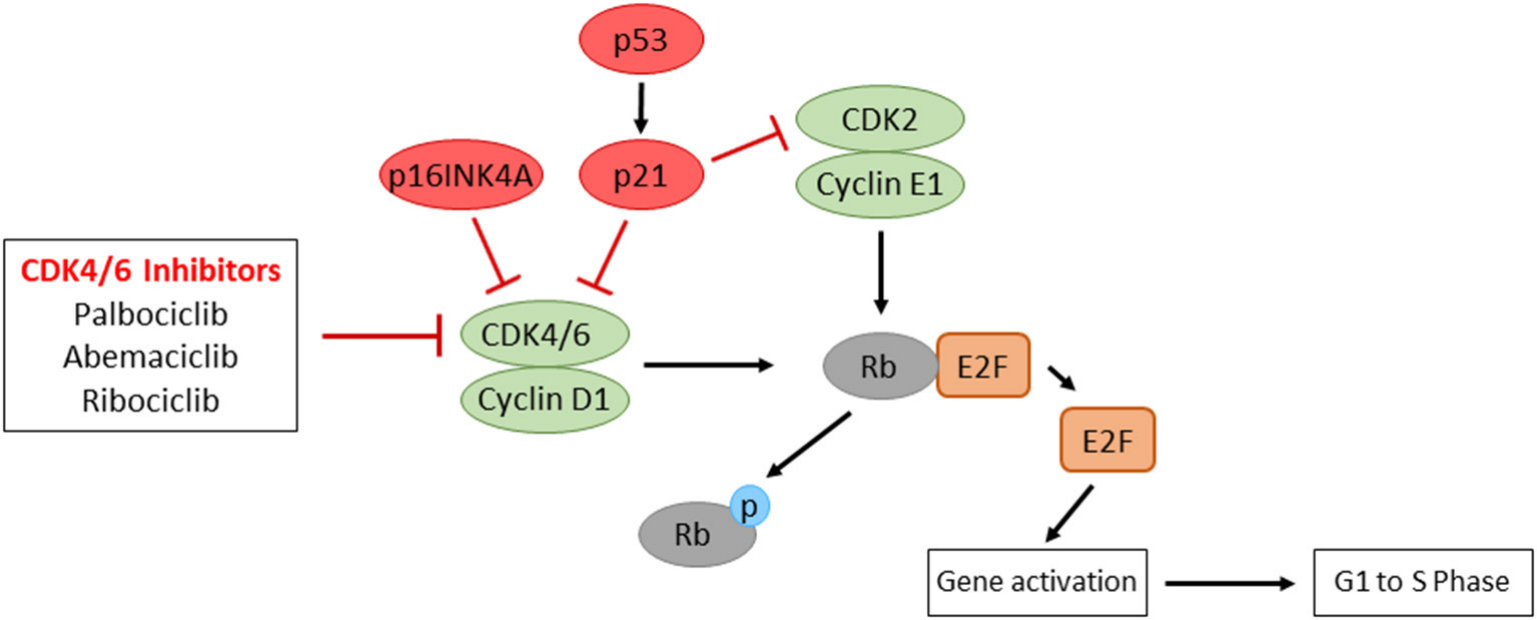
The representative regulatory pathways of cell cycle progression and the mode of action of CDK4/6 inhibitors. Overexpression of Cyclin D1, inactivation of p16INK4A, or inactivated mutations of Rb, which are often observed in HNSCC patients, contribute to uncontrolled cell proliferation. CDK4/6 inhibitors result in the inhibition of E2F transcriptional activity, which show promising prognostic outcomes in HNSCC patients.

In HPV(-) HNSCC, the cyclin D–CDK4/6–INK4–Rb pathway is impaired by amplification of *CCND1* (20-30% of cases) and inactivation of *p16INK4A* (approximately 90% of cases) [1, 68]. *RB1* is also found to be impaired in ~5% of patients. In HPV(+) HNSCC, E7 protein directly binds to Rb, resulting in proteasomal degradation of Rb to release E2F. Since the CDK4/6 activity is important for the regulation of cell proliferation, the selective inhibition of CDK4/6 appears as a promising therapeutic choice (Figure 4). Several clinical trials to inhibit CDK4/6 activity have been/are being performed in recurrent/metastatic HNSCC. A recent phase II trial with cisplatin-resistant or cetuximab-resistant HPV(-) HNSCC patients treated with a CDK4/6 inhibitor, palbociclib, and cetuximab results in promising activity outcomes [135]. A phase I trial with recurrent/metastatic p16-negative HNSCC patients treated with a CDK4/6/inhibitor, ribociclib, and cetuximab showed safety and efficacy [136]. With a CDK4/6 inhibitor, abemaciclib, phase II trials are underway evaluating the effects of abemaciclib monotherapy or abemaciclib plus nivolumab in HPV(–) HNSCC (NCT03655444, NCT04169074). These studies will elucidate whether CDK4/6 inhibitors play a therapeutic role in the prognostically unfavorable p16/HPV(–) subgroup of patients.

## Conclusion

Treatment for HNSCC is still generally multimodal, consisting of surgery followed by chemoradiation for oral cavity cancers and primary chemoradiation for pharynx and larynx cancers. It is obvious that HNSCC are able to use a multitude of mechanisms to develop treatment resistance. Thus, one single strategy might not be sufficient, and increased insights to the resistance mechanisms as well as identification of biomarkers will help to design novel and optimized therapeutic strategies, which either direct against tumor cells and/or modulate tumor microenvironment. These issues are also discussed in several recent excellent reviews [3, 137–139].

## Author Contributions

TB and HH wrote the initial draft of the manuscript. JAR made substantial revision. All authors contributed to manuscript revision and approved the final version.

## Conflict of Interest

JAR is a part of Light Switch Bio, LLC., a startup company that is developing light-based therapeutics and diagnostics for commercial applications, including the treatment of cancers. The remaining authors declare that the research was conducted in the absence of any commercial or financial relationships that could be construed as a potential conflict of interest.
